# Supplementary material for: Activation of Serum/Glucocorticoid Regulated Kinase 1/Nuclear Factor-κB Pathway Are Correlated with Low Sensitivity to Bortezomib and Ixazomib in Resistant Multiple Myeloma Cells
Source: Biomedicines. 2021 Jan 4;9(1):33. doi: 10.3390/biomedicines9010033 (PMC7823718; doi:10.3390/biomedicines9010033)

Supplementary Figure

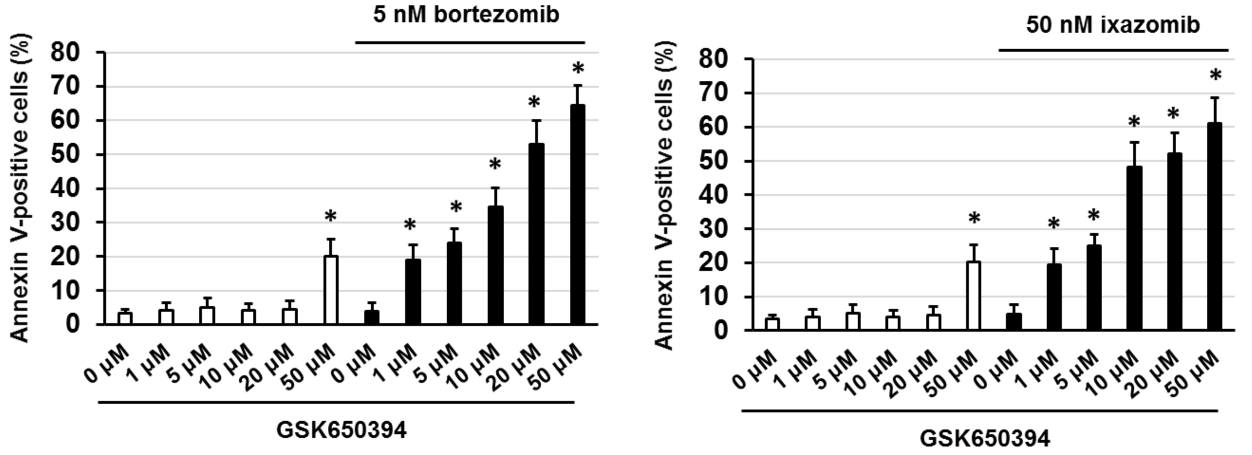

Figure S1. Combined effect of GSK650394 and bortezomib or ixazomib on Annexin-V positive cells in KMS-20 cells, evaluated by Muse™ Annexin V and Dead Cell kit. Annexin-positive cells of GSK650394- and bortezomib- or ixazomib-treated KMS-20 cells as measured by the Muse™ Annexin V and Dead Cell kit (Merck Millipore, Nottingham, UK). These cells were administrated with indicated concentrations of GSK650394 and bortezomib or ixazomib for 48 hrs. Then, cells washed twice phosphate buffered saline, were mixed with Muse™ Annexin V and Dead Cell Assay Kit reagents. Analysis were performed using a Muse Cell Analyzer. The results showed the 5 independent experiments. *p < 0.01 vs. untreated cells (Shapiro-Wilk test and one-way analysis of variance (ANOVA) with Dunnett's test.).

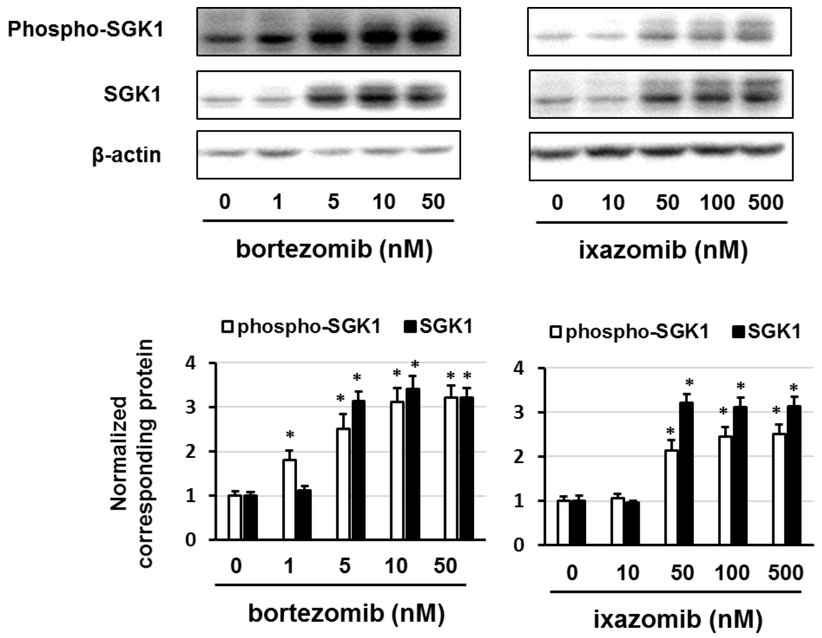

Figure S2. Effect of bortezomib and ixazomib on phosphorylated SGK1 and total SGK1 expression in KMS-20 cells. KMS20 cells were treated with bortezomib and ixazomib for 3 day. The cell lysates were examined by western blotting using the indicated antibodies. Quantification of the amount of phosphorylated SGK1 and total SGK1, normalized to the amounts of β-actin. The results are representative of three independent experiments. * p < 0.01, compared to controls (one-way ANOVA with Dunnett's test).

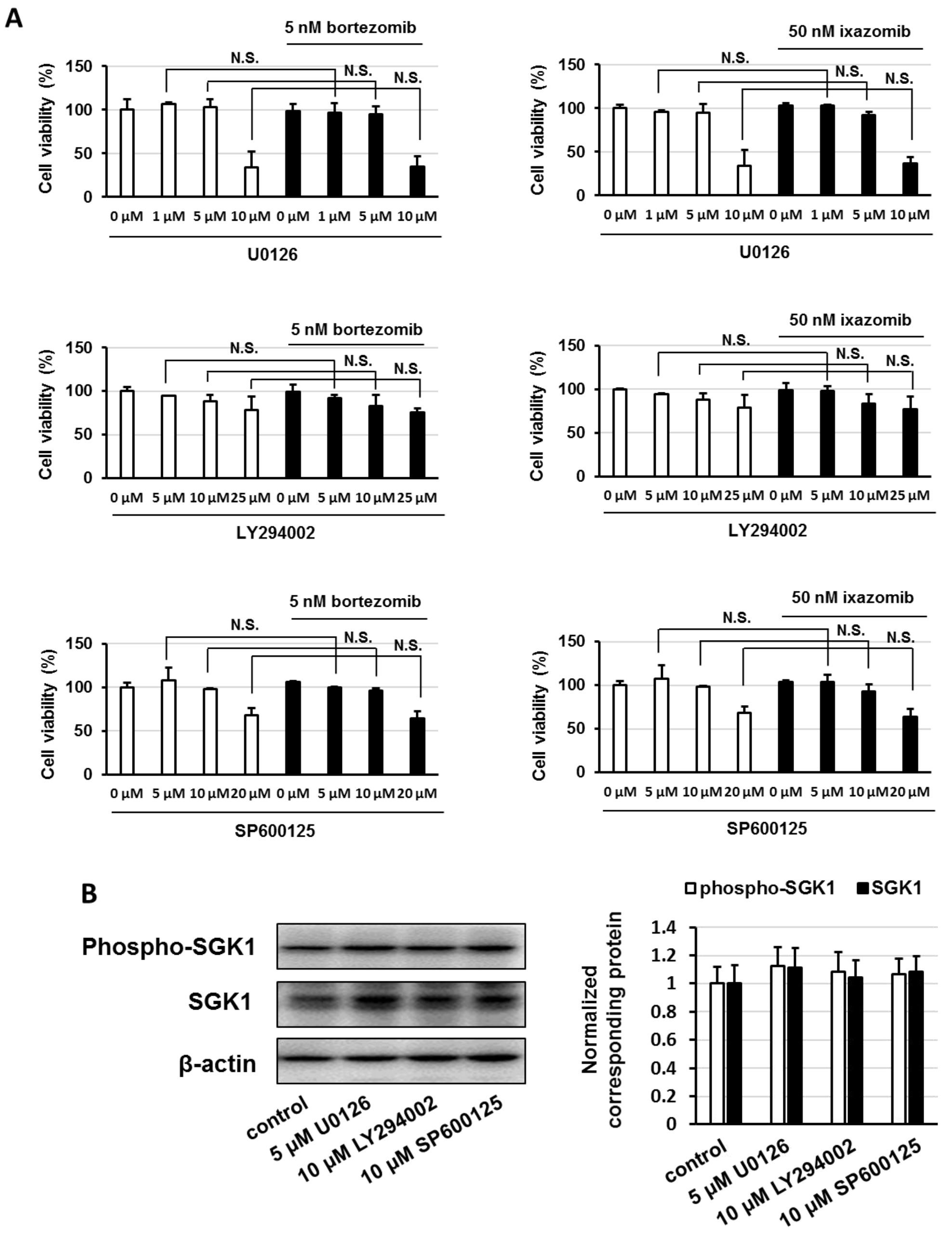

Figure S3. Effect of U0126, LY294002, and SP600125 on bortezomib or ixazomb resistance. (A) KMS-20 cells were administered the indicated concentrations of bortezomib, ixazomib, U0126, LY294002, or SP600125. After incubation for 72 h, cell viability was analyzed by trypan blue staining. The results are representative of five independent experiments. * $p < 0.01$ vs. untreated cells, as assessed with the Shapiro-Wilk test and one-way analysis of variance (ANOVA) with Dunnett's test. (B) Cell lysates were examined by western blotting using the indicated antibodies. Quantification of the amount of phosphorylated SGK1 and total SGK1, normalized to the amounts of β-actin. The results are representative of three independent experiments.

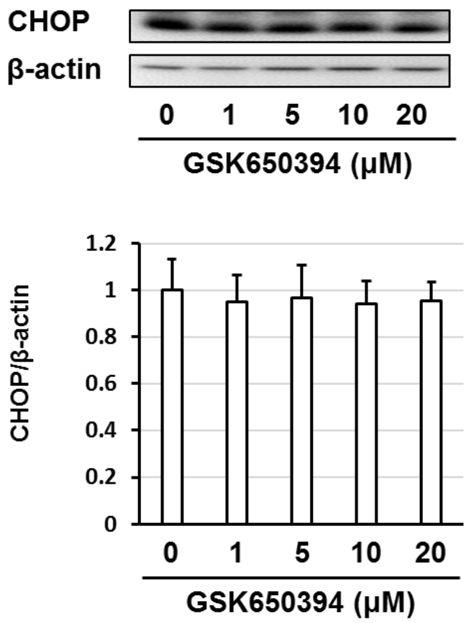

Figure S4. Effect of GSK650394 on CHOP expression in KMS-20 cells. KMS20 cells were treated with GSK650394 for 3 day. The cell lysates were examined by western blotting using the indicated antibodies. Quantification of the amount of CHOP, normalized to the amounts of β-actin. The results are representative of three independent experiments.

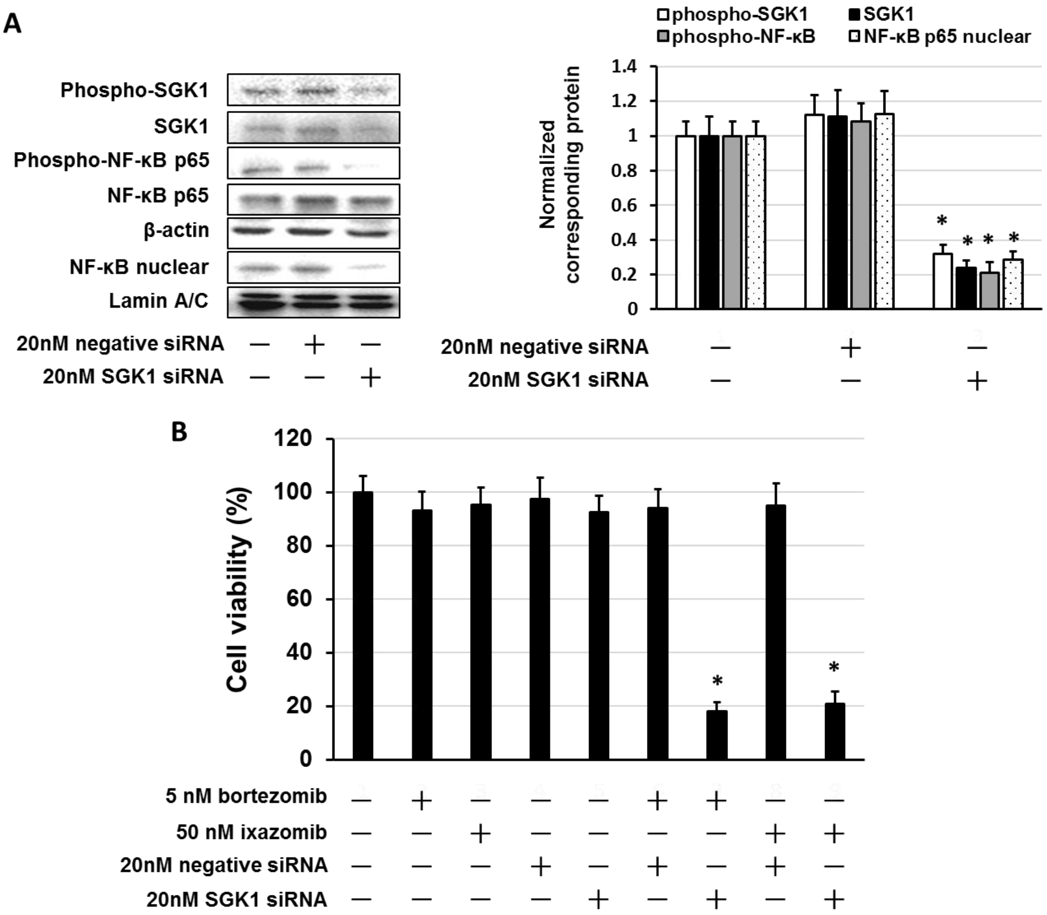

Figure S5. Effect of SGK1 siRNA on bortezomib or ixazomb resistance. The double-stranded small interfering RNAs (siRNAs) targeting SGK1 (HSS106477) were synthesized and purified by Invitrogen (Carlsbad, CA, USA). StealthTM RNAi negative control duplex (low GC content) (Invitrogen) was used as a negative control. Transfection of siRNAs was performed according to the manufacturer's protocol by using the LipofectamineTM 3000 reagent (Invitrogen). Briefly, 4 µl of 20-µM siRNA was mixed with 200 µl of Opti-minimum essential medium (MEM®). LipofectamineTM 3000 (4 µl) was diluted in 200 µl of Opti-MEM® and incubated at room temperature for 5 min. After incubation, the diluted LipofectamineTM 3000 was mixed with the diluted siRNA and further incubated for 20 min at room temperature. In total, 400 µl of the siRNA-LipofectamineTM 3000 complex was applied to each well containing cultured KMS-20 cells at approximately 50–70% confluence in 6-well microplates. (A) KMS-20 cells were treated with SGK1 siRNA, or a negative control siRNA for 1 day. Control cells were treated with PBS for 3 days. The cell lysates were examined by western blotting using the indicated antibodies. Quantification of the amount of phospho-SGK1, SGK1, phospho-NF-κB p65, or NF-κB p65 nuclear, normalized to the amounts of NF-κB, β-actin, or Lamin A/C. The results are representative of three independent experiments. * $p < 0.01$, compared to controls (one-way ANOVA with Dunnett's test). (B) KMS-20 cells were exposed to the indicated concentrations of SGK1 siRNA, negative siRNA, bortezomib or ixazomib. After incubation for 72 h, the number of dead cells was counted by trypan blue staining. The results are representative of 5 independent experiments. *$p < 0.01$ vs. untreated KMS-20 cells (Shapiro-Wilk test and one-way analysis of variance (ANOVA) with Dunnett's test.).

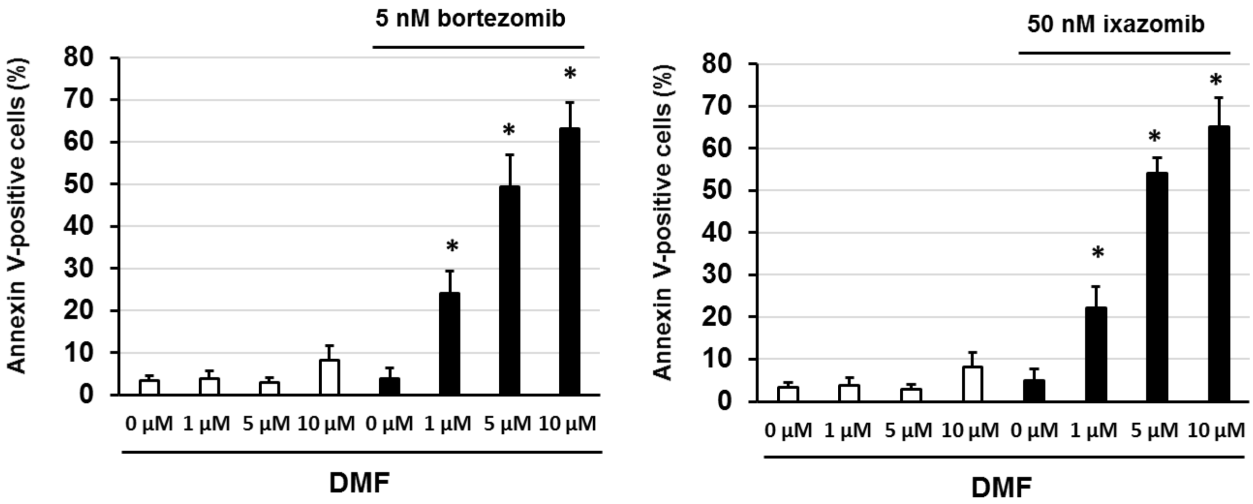

Figure S6. Combined effect of dimethyl fumarate (DMF) and bortezomib or ixazomib on Annexin-V positive cells in KMS-20 cells, evaluated by Muse™ Annexin V and Dead Cell kit. Annexin-positive cells of DMF- and bortezomib- or ixazomib-treated KMS-20 cells as measured by the Muse™ Annexin V and Dead Cell kit (Merck Millipore, Nottingham, UK). These cells were administrated with indicated concentrations of DMF and bortezomib or ixazomib for 48 hrs. Then, cells washed twice phosphate buffered saline, were mixed with Muse™ Annexin V and Dead Cell Assay Kit reagents. Analysis were performed using a Muse Cell Analyzer. The results showed the 5 independent experiments. *$p < 0.01$ vs. untreated cells (Shapiro-Wilk test and one-way analysis of variance (ANOVA) with Dunnett's test.).

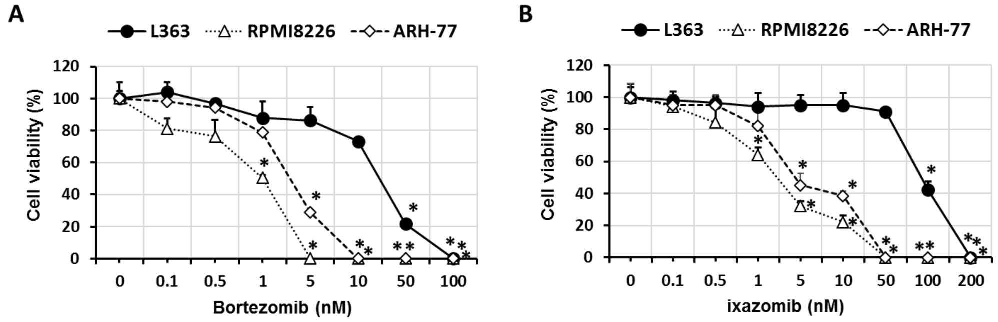

C

Cell line	IC50 (nM)	
	bortezomib	ixazomib
L363	28.5	91.2
ARH-77	2.2	4.1
RPMI8226	1.2	2.7

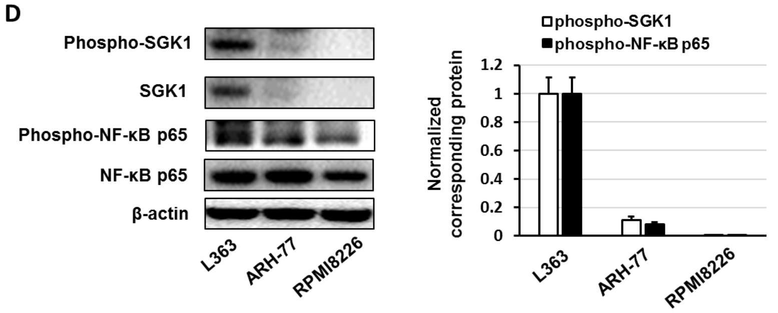

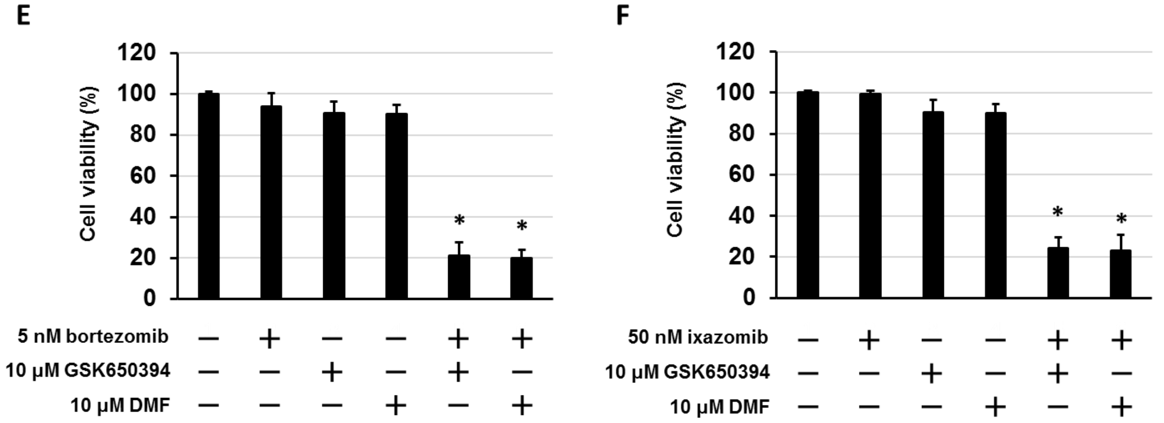

Figure S7. Effect of bortezomib and ixazomib on L363, ARH-77, and RPMI8226 cell viability. L363 cells were obtained from the German Collection of Microorganisms and Cell Cultures (DSMZ, Braunschweig, Germany). RPMI8226 cells were obtained from Japanese Collection of Research Resources Cell Bank (Osaka, Japan). ARH77 cells were obtained from DS Pharma Biomedical (Osaka, Japan). These cells were maintained in RPMI 1640 medium (Sigma, St. Louis, MO, USA) supplemented with 25 mM of 4-(2-hydroxyethyl)-1-piperazineethanesulfonic acid (pH 7.4; FUJIFILM Wako), 100 U/ml streptomycin (Gibco, Carlsbad, CA, USA), 100 μg/ml penicillin (Gibco), and 10% fetal bovine serum (Gibco) in an atmosphere of 5% CO_2. Viability of (A) bortezomib- and (B) ixazomib-treated L363, ARH-77, and RPMI8226 cells as measured by the trypan blue dye assay. These cells were treated with the indicated concentrations of bortezomib for 3 days. The results are representative of five independent experiments. * $p < 0.01$ vs. controls (Shapiro-Wilk and Kruskal-Wallis tests, followed by the Steel test.). (C) IC50 of bortezomib and ixazomib for L363, ARH-77, and RPMI8226 cells. (D) Cell lysates were examined by western blotting using the indicated antibodies. Quantification of the amount of phospho-SGK1 or phospho-NF-κB p65, normalized to the amounts of SGK1 or NF-κB. The results are representative of three independent experiments. * $p < 0.01$, compared to controls

(one-way ANOVA with Dunnett's test). (E, F) L363 cells were administered the indicated concentrations of (E) bortezomib, (F) ixazomib, GSK650394, or DMF. After incubation for 72 h, cell viability was analyzed by trypan blue staining. The results are representative of five independent experiments. * $p < 0.01$ vs. untreated cells, as assessed with the Shapiro-Wilk test and one-way analysis of variance (ANOVA) with Dunnett's test.

Figure 2A

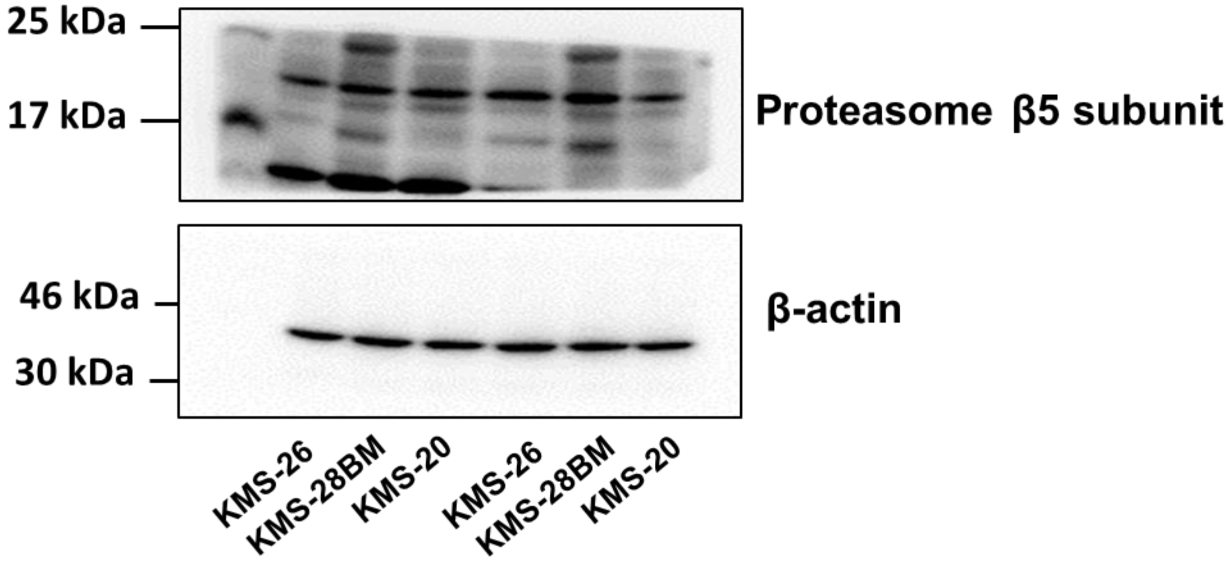

Figure 3B

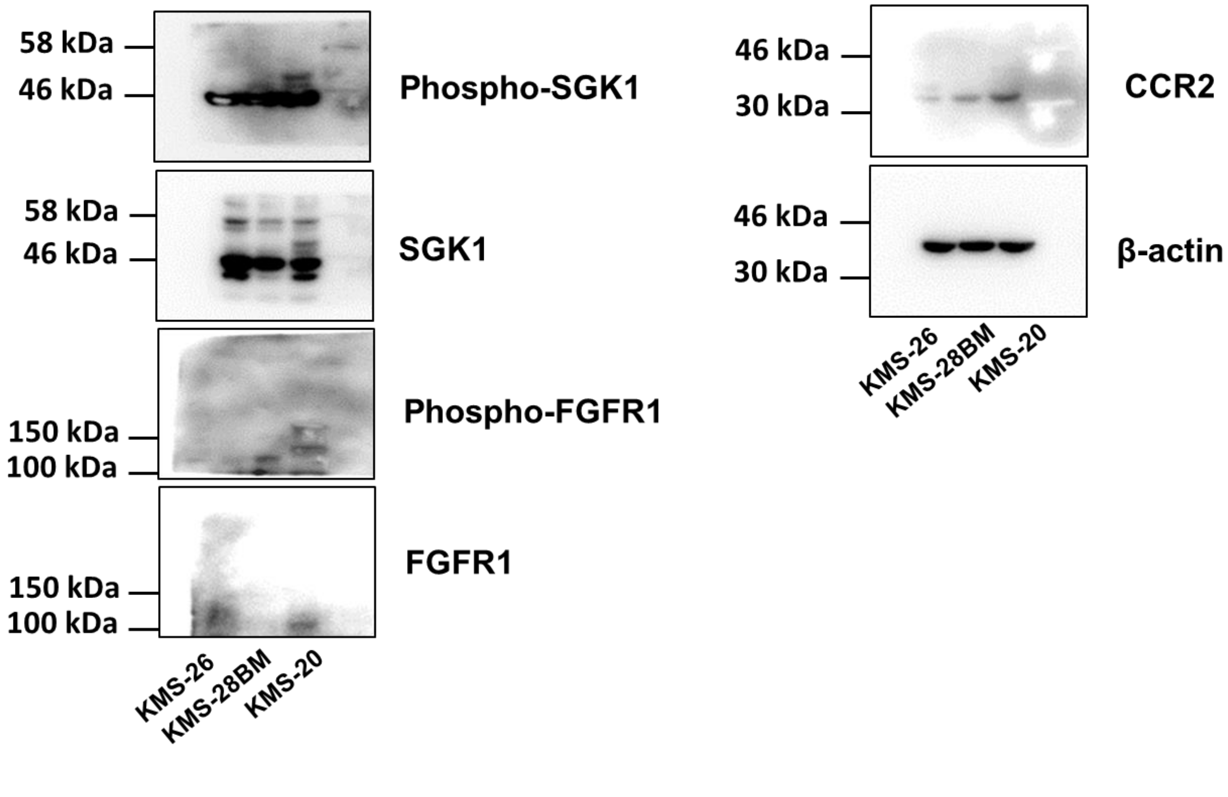

Figure 4B

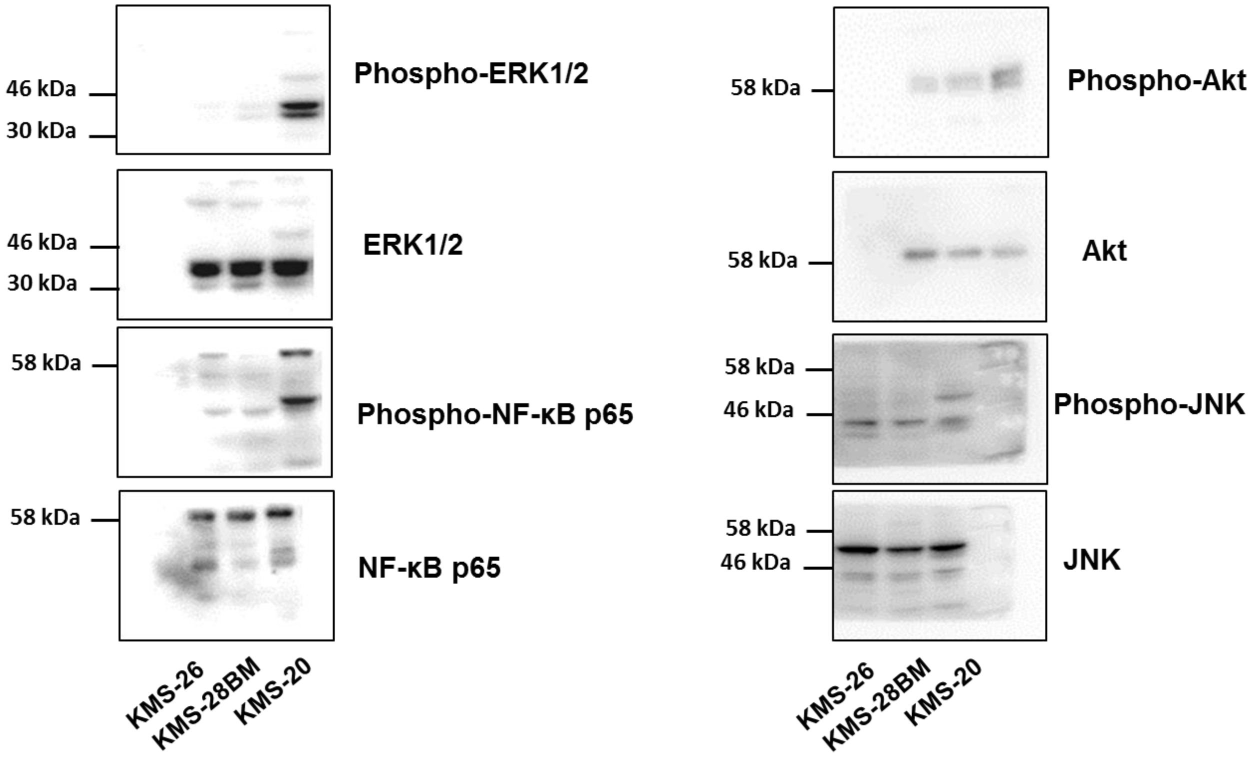

Figure 4C

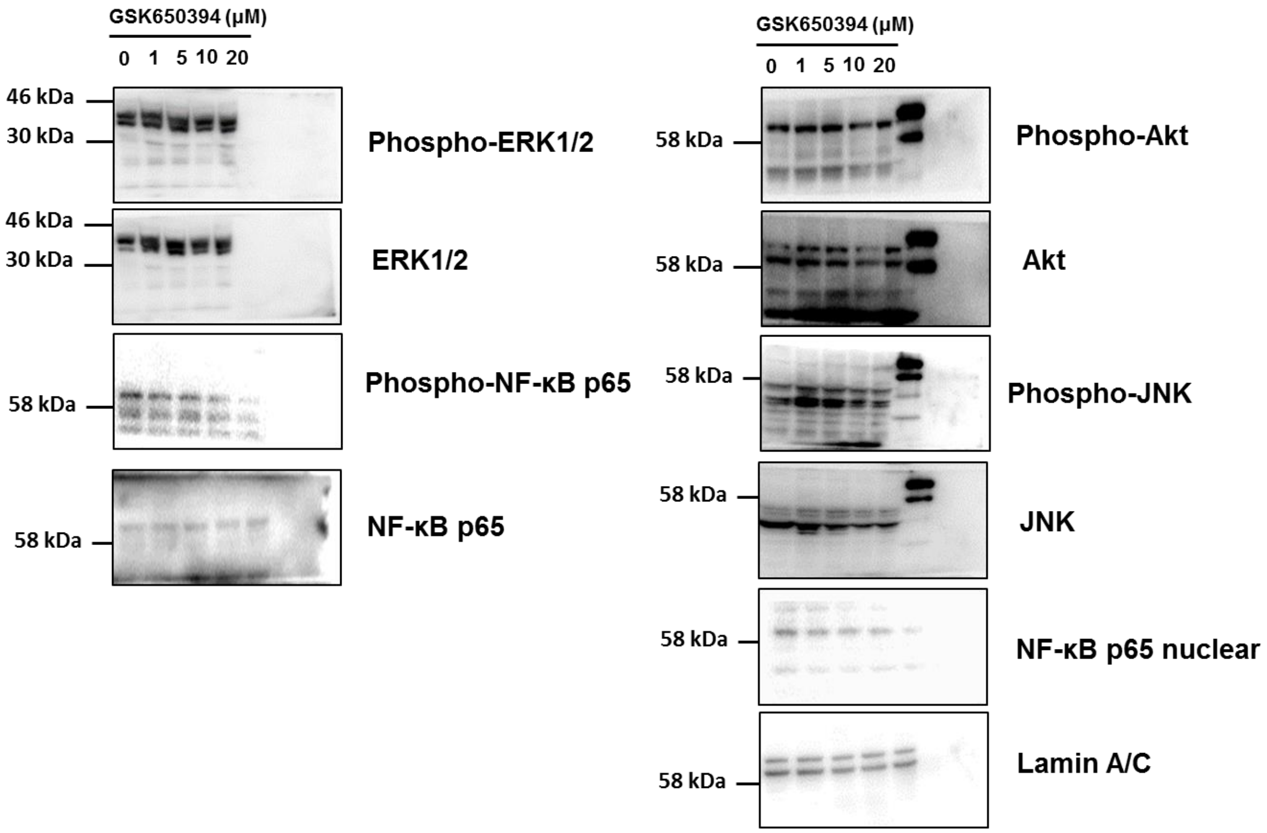

Figure 4D

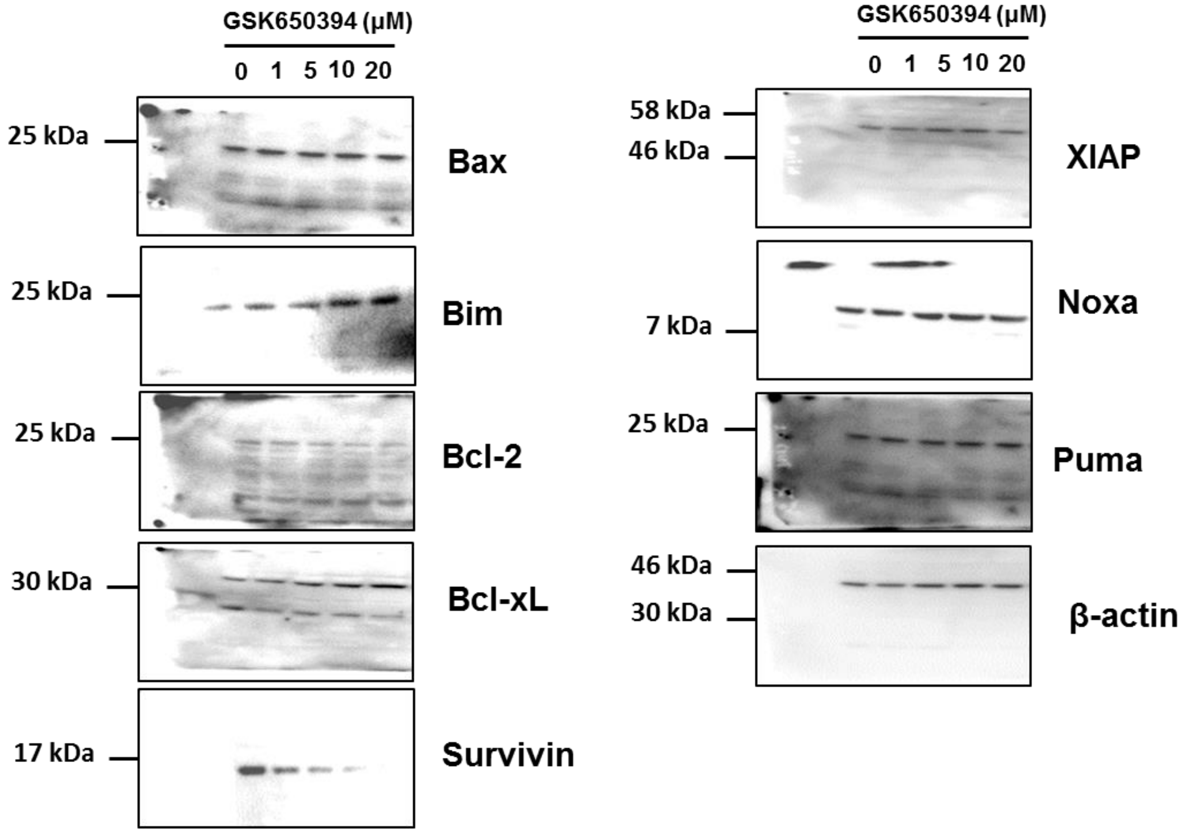

Figure 5B

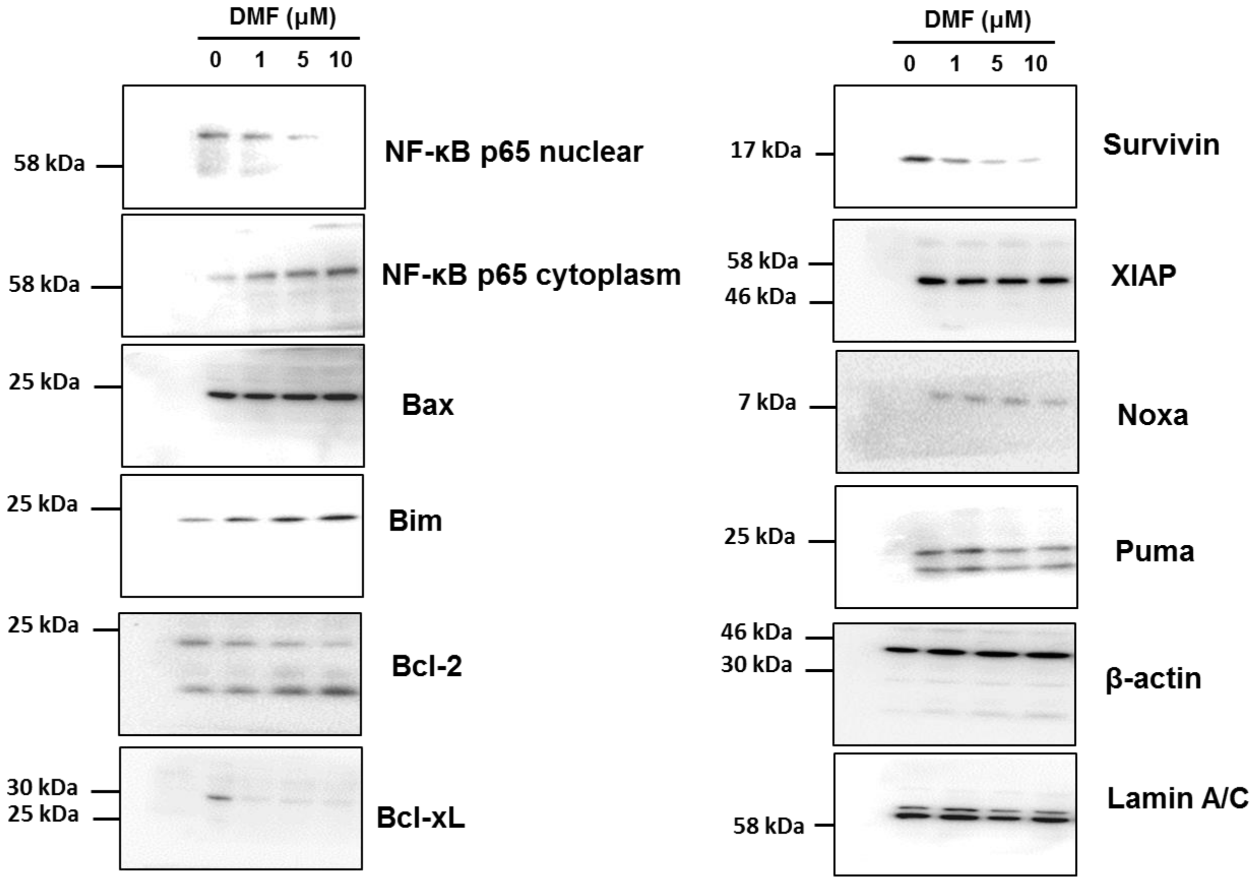

Supplementary Figure 2

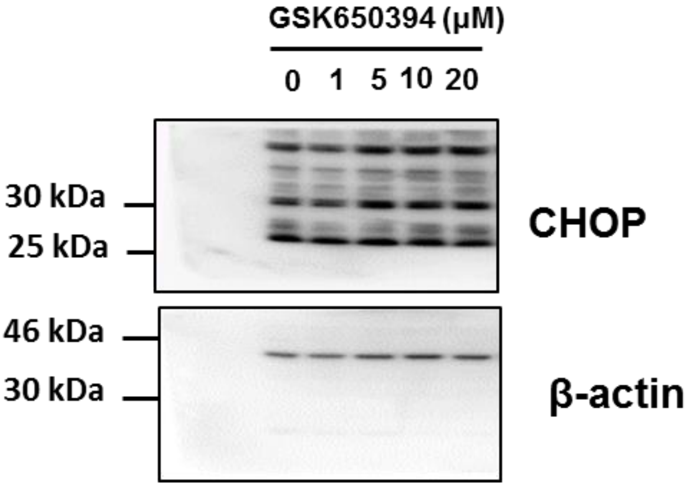

Supplementary Figure 3A

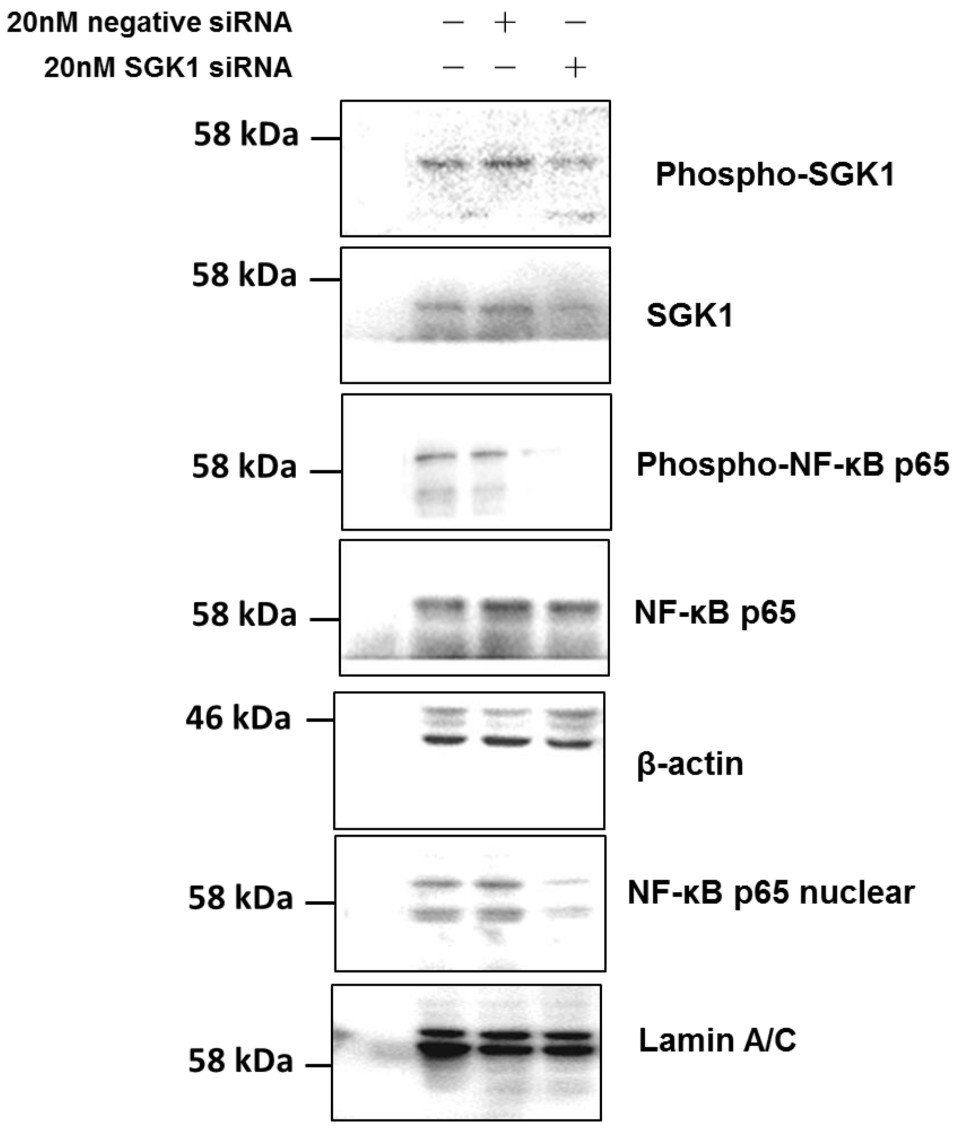

Supplementary Figure 5B

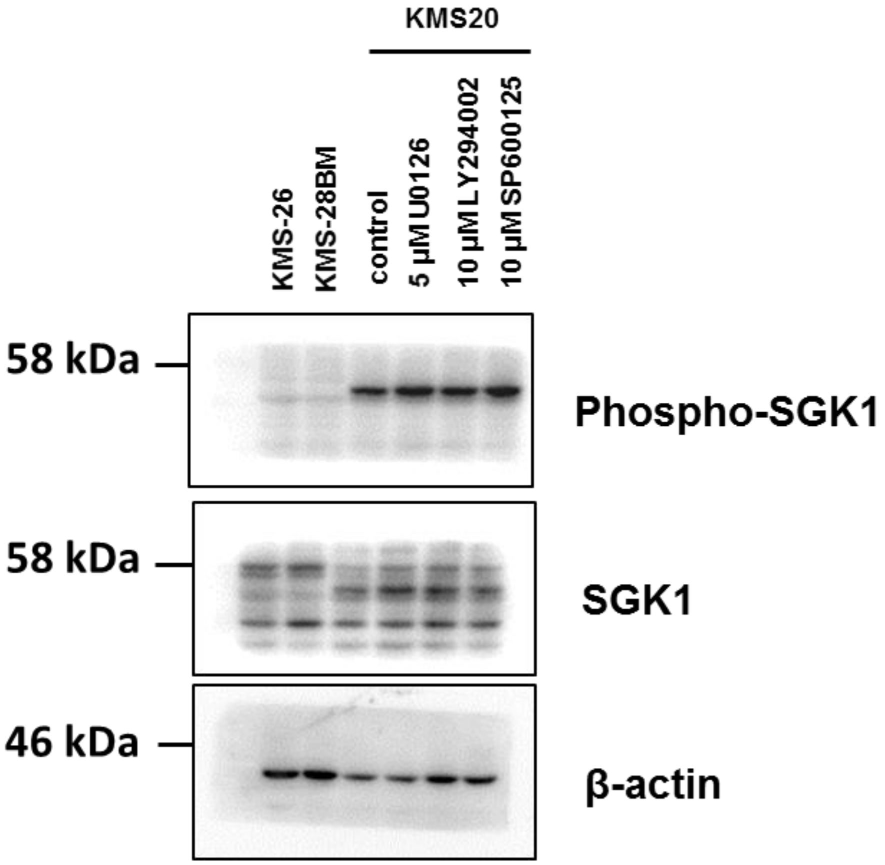

Supplementary Figure 6D

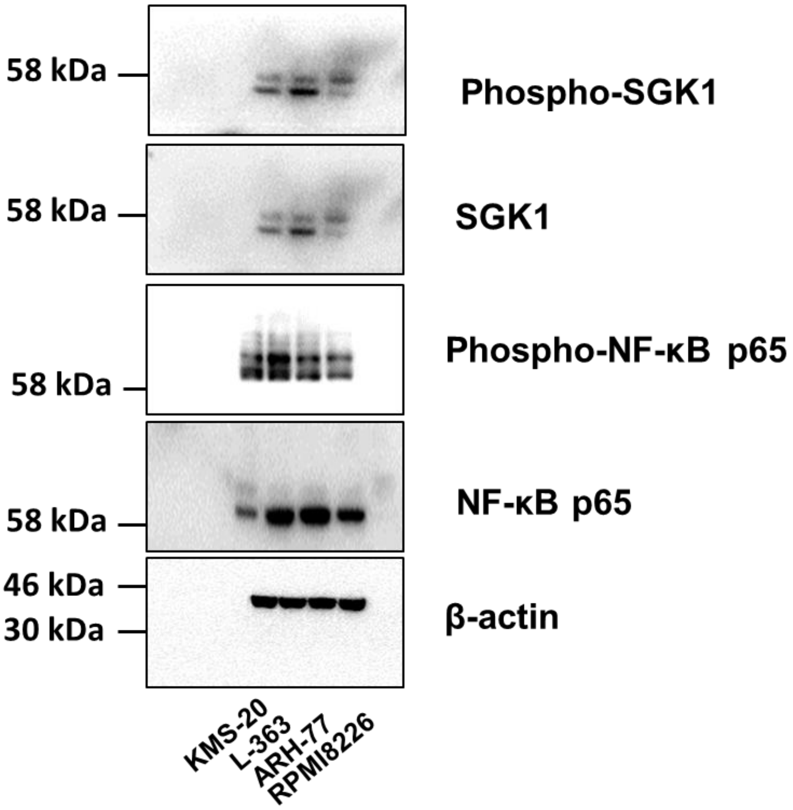

Supplement: Supplementary file 1 [file biomedicines-09-00033-s001.pdf]
